# Depressive risk among Italian socioeconomically disadvantaged children and adolescents during COVID-19 pandemic: a cross-sectional online survey

**DOI:** 10.1186/s13052-022-01266-x

**Published:** 2022-05-07

**Authors:** Maria Serra, Anna Presicci, Luigi Quaranta, Maria Rosaria Erminia Urbano, Lucia Marzulli, Emilia Matera, Francesco Margari, Lucia Margari

**Affiliations:** 1 Department of Pharmacy-Pharmaceutical Sciences, University of Bari “Aldo Moro”, Via Edoardo Orabona 4, Bari, Italy; 2 Department of Neuroscience, Sense Organs and Locomotor System, University Hospital “Policlinico”, Piazza Giulio Cesare 11, 70124 Bari, Italy; 3 Department of Computer Science, University of Bari “Aldo Moro”, Via Edoardo Orabona 4, Bari, Italy; 4 Department of Biomedical Sciences and Human Oncology, University of Bari “Aldo Moro”, Piazza Giulio Cesare 11, 70124 Bari, Italy; 5 Department of Basic Medical Science, Neuroscience and Sensory Organs, University of Bari “Aldo Moro”, Piazza Giulio Cesare 11, 70124 Bari, Italy

**Keywords:** COVID-19, Socioeconomic disadvantage, Low-income, Children, Adolescents, Depression, Mental health

## Abstract

**Background:**

Children and adolescents and low-income individuals are considered particularly vulnerable for mental health implications during the current COVID-19 pandemic. Depression is a frequent negative emotional response during an epidemic outbreak and is also prone importantly to environmental risk like stressors derived from income inequality. We aimed to assess depressive symptomatology in a sample of Italian low-income minors during the COVID-19 outbreak. We hypothesized that the stronger were the negative effects of the pandemic on socioeconomic conditions, the higher would have been the risk for showing depressive symptoms.

**Methods:**

We performed a cross-sectional study during July 2020, at the end of the Italian first wave of COVID-19 pandemic. We recruited 109 Italian socioeconomically disadvantaged children and adolescents from 7 to 17 years. We used an online survey to collect socio-demographic and clinical data and information about pandemic-related stressors and to assess depressive symptoms with the Children’s Depression Inventory 2 (CDI 2), Parent Version (Emotional Problems subscale) and Self-Report Short Form. We performed logistic regression analysis to assess the association between depressive symptoms and potential risk factors for mental health.

**Results:**

22% and 14% of participants showed depressive symptoms at the CDI 2 Parent Version and Self-Report, respectively. Participants coming from families experiencing a lack of basic supplies during the pandemic (34.9%) were more expected to show depressive symptoms at CDI 2 Parent Version. Participants with a pre-existing neuropsychiatric diagnosis (26.6%) were more likely to exhibit depressive symptoms measured by CDI 2 Parent Version.

**Conclusions:**

The results of our study showed that a group of Italian socioeconomically disadvantaged children and adolescents were more vulnerable to depressive symptoms if they suffered from a paucity of essential supplies during the pandemic or had pre-existing neurodevelopmental disorders. The promotion of educational and child-care programs and activities could be crucial in sustaining the prevention of mental distress in those frail subjects who particularly need support outside the family. Further studies are needed to detect effective preventive and therapeutic strategies to adopt promptly in the case of another pandemic wave.

## Background

The World Health Organization declared Coronavirus Disease 2019 (COVID-19) a pandemic on March 12th, 2020 [1]. Italy was the first European country to face this new health emergency and has been harmed, to date, by four separate waves of the pandemic (during spring and autumn 2020, spring 2021, and winter 2021–2022).

During the COVID-19 outbreak, both the pandemic and containment measures taken by the governments have profoundly impacted daily life [2, 3]. Isolation, contact restriction, and economic shutdown have generated dramatic psychological, social, and economic consequences [2, 4].

Since the beginning of the pandemic, children and young people have been considered one of the frailest groups for mental health implications, having been affected by school closure, heightened exposure to domestic violence and child maltreatment, and change and disruption of social networks [5]. Among them, particularly vulnerable are those belonging to low-income households, as the paucity of essential supplies under normal conditions are exacerbated during pandemics. The necessary home confinement, ordered by the government authorities, could have led to a forced interruption of the temporary works of their parents, parental unemployment, and financial insecurity.

Among the containment measures, school closure has affected millions of children worldwide and has had a significant impact on children and adolescents, especially on socioeconomically disadvantaged ones [4, 5, 7–9]. The necessary shift to the home-based distance learning models has meant the absence of school routines and outdoor activities, reduced social contact, and dropping education [6, 7, 10, 11]. The potentially negative implications of similar modifications on the psychological balance of children and adolescents could be even more pronounced for those living in poverty [6, 7, 10, 11]. They often do not own an electronic device or Internet connections, making homeschooling challenging to take part in [4, 5, 8].

In Bari province, in the South of Italy, Diurnal Socio-Educational Centers (DSECs) support low-income families in the afternoon post-school period, working as semi-residential structures. The DSECs belong to the territorial social services network offering holistic support: they operate in close connection with the school, the social services, and other educational structures; ensure educational support in learning activities; enhance good personal hygiene; contribute to healthy free nutrition by giving the midday meal and a snack; provide parenting support to the children’s families [12]. A few days after the school closure (March 8th, 2020, throughout Italy), on March 12th also the DSECs were closed, as determined by the civil authorities for the entire regional territory to which Bari belongs. Short-term consequences were a further reduction of social contacts and educational support besides the loss of free healthy nutrition. The socioeconomically disadvantaged families enjoying that service found themselves trying to sustain their children through their scarce resources**.** In this way, the DSECs shutdown increased the already heavy load given by school closure.

If poverty is a well-known risk factor for mental health in children and adolescents, the socio-ecological impact of the pandemic could increase this pre-existing vulnerability, both in the short term and for a lifetime [2]. It is especially true for those mental health conditions, such as depression, that are more prone to environmental risk, like stressors derived from income inequality [13, 14]. One of the research priorities during the current pandemic was monitoring and reporting rates of depression and other mental health problems across the general population and vulnerable groups [2, 5]. Although lower socioeconomic conditions have been often addressed by literature as a risk factor for children’s mental health during COVID-19, only a few original studies have approached this subject, to our knowledge. Findings from the early phases of the pandemic demonstrated more pronounced behavioral and anxiety problems among young children (5 years old or less) from economically disadvantaged households [15, 16]. A large cross-sectional Chinese study found a higher risk for psychosocial problems in children (2–12 years old) from low-income families [17]. In a recent Italian study, adolescents living in unfavorable environmental conditions were more prone to suffer from depression during the full Italian lockdown [18].

The novelty of our study was to consider the increased vulnerability for the mental health of young people who are socially and economically deprived, represented by minors attending DSECs. This research has aimed to determine whether the COVID-19 pandemic and its containment measures were predictors of depressive symptoms in such a population. We recruited a sample of unprivileged children and adolescents attending the DSECs of the Bari province in the South of Italy. We investigated depressive symptoms at the end of the first wave of the pandemic (that in Italy covered the months of March and April 2020, with a slow return to normality during May and June 2020). We hypothesized that in a population at risk for depression as the socioeconomically disadvantaged children and adolescents, the stronger were the negative effects of the pandemic on socioeconomic conditions (e.g., food insecurity, parental unemployment, impaired access to homeschooling), the higher would have been the risk for showing depressive symptoms. Furthermore, we assessed if other pandemic-related stressors (i.e., Sars-CoV-2 infection, quarantine) and pre-existing neuropsychiatric diagnoses would behave as risk factors for depressive symptoms.

## Methods

### Setting and participants

This study was a cross-sectional online survey created on Google Forms. The questionnaire was addressed to 7 to 17-year-old individuals living in the Bari province, attending a DSEC of the territory because of their socioeconomic disadvantage. The DSECs are places of social and cultural integration for 6 to 17-year-old individuals with social problems and exposed to the risk of marginalization or deviance [12]. These structures are regulated by the regional legislation [19]: only people coming from a low socioeconomic level can access, given the required certification of an Equivalent Economic Status Indicator lower than 20.000,00 Euros [12]. We applied to the 48 DSECs of the Bari province by telephone call and an invitation letter by email, requesting their availability to participate. We sent the survey's link to the managers of the 19 DSECs who declared themselves available. The educational team of each DSEC proposed, explained, and transmitted the survey to the parents of children and adolescents enjoying the service. Overall, 109 participants took part in the study. Our sample size exceeds 91, i.e., the size of a representative sample of the studied population with a 10% margin of error and 95% confidence interval. Such population comprises all individuals attending a DSEC in the province of Bari. Their number can be roughly estimated to 1500, considering that each of the 48 DSECs hosts about 30 minors. The form was administered from July 14th to July 29th, 2020, at the end of the Italian first wave of COVID-19. We included our email addresses at the beginning of the questionnaire to facilitate any consultation. The minors filled out the survey after their parents had consented. Anonymous data were collected.

### Measurements

The online questionnaire assessed the point of view of both the parents and their children with questions concerning general information, variables related to the COVID-19 pandemic, and depressive symptoms measured by the Children’s Depression Inventory 2 (CDI 2). The original version of the CDI 2 was developed in 2011 by Maria Kovacs; we used the Italian version, developed and validated by the authors Camuffo and Cerutti in 2017 [20]. The online questionnaire presented the following sections:

#### Parent-report

- Socio-demographic and clinical characteristics: sex, age, municipality of residence, attendance to a territorial DSEC, pre-existing neuropsychiatric diagnoses.
- Pandemic-related stressors: Sars-CoV-2 infection (affecting the child and/or a family member); mandatory quarantine (affecting the child and/or a family member); impact on the parental job (i.e., no access to previous occupation, layoff, dismissal, no changes); impact on the availability of basic supplies (i.e., difficulty in finding basic supplies, more material help received than before); impact on access to homeschooling (i.e., homeschooling from the beginning or within one month or beyond one month, no homeschooling at all, no homeschooling due to unavailability of devices).
- Depressive symptoms, assessed by the Italian version of the CDI 2, Parent Version (PV). It is a 17-item questionnaire based on a 4-point Likert scale (0–3), to be completed by the parents of young people aged 7–17 years. It gives information about children’s depressive symptoms, in the last two weeks, from the parental point of view. The CDI 2 PV provides two partial scores: Emotional Problems (related to dysphoric feelings and neurovegetative symptoms) and Functional Problems (related to the functional consequences on family, school, and social contacts); the total score of the questionnaire is given by the sum of the two partial scores. In this study only the Emotional Problems score was considered, as the Functional Problems score would have been highly influenced by the lack of school attendance during the summer period: indeed, 3 out of the 8 items contributing to the latter partial score explicitly refer to school. The psychometric characteristics of the Italian version of the CDI 2 PV, Emotional Problems subscale, show high validity and reliability, with internal consistency values ranging from 0.61 and 0.74 in a normative sample of Italian students and 0.89 in a clinical sample [20]. In this study, Cronbach’s alpha was 0.73. T-scores normed based on age and sex were used in the present study. T-scores ≥ 60 were considered positive for depressive symptoms, as per the CDI 2 scoring guidelines [20].

#### Self-report by children and adolescents

- Major difficulties perceived by minors, through a question developed by our research team (“What do you think was the greatest difficulty for you to face in this period?”). More than one answer was possible among: fear of getting sick or my relatives and friends getting sick; not being able to go to school; not being able to go to the DSEC; concern for the work of mum and dad; not having enough water or food or clothing; failing to access homeschooling due to the lack of a computer or a tablet or internet connection.
- Depressive symptoms, assessed by the CDI 2 Self-Report (S-R) Short Form (SF). It is a 12-item 3-point (0–2) Likert’s self-report scale, widely used for screening purposes to investigate children’s depressive symptoms, in the last two weeks, from a subjective point of view. It provides a single total score. The psychometric characteristics of the Italian version of the CDI 2 S-R SF show high validity and reliability, with internal consistency values ranging from 0.68 to 0.78 in a normative sample of Italian students and 0.80 in a clinical sample [20]. In this study, Cronbach’s alpha was 0.71. The cut-off for depressive symptoms was set at T-scores ≥ 60.

### Statistical analysis

Data were analyzed using “pandas” and “statsmodels”: two software libraries written for the Python programming language for data manipulation and statistical analysis. Computation results and data visualizations were collected and documented in Python Jupyter notebooks. We used descriptive statistics and frequency analysis to analyze: socio-demographic data; pandemic-related stressors; the major difficulties perceived by children and adolescents; the depressive symptoms. We performed logistic regression analysis to assess the association between depressive symptoms and potential risk factors for mental health (pre-existing neuropsychiatric diagnoses and pandemic-related stressors). The statistical significance level was set at *p* < 0.05.

## Results

### Participants socio-demographic and clinical characteristics

The sample size of our survey included 109 participants (M: F = 1.6: 1), from 7 to 17 years old. The median age was 12 years. The whole sample was resident in the Bari Province and attended a territorial DSEC, as requested to be recruited in the survey. Therefore, all the participants had a socioeconomic disadvantage. A pre-existing neuropsychiatric diagnosis was present in 29 subjects (26.6%), most frequently represented by Specific Learning Disability (SLD) (12.8%) and Attention-Deficit/Hyperactivity Disorder (ADHD) (8.3%). Table 1 reports complete socio-demographic and clinical characteristics.

**Table 1:** Sample socio-demographic and clinical characteristics

Variables	N (%)
Participants	109
Sex	
Male	68 (62.4%)
Female	41 (37.6%)
Age	
7–12 years	65 (59.6%)
13–17 years	44 (40.4%)
Pre-existing neuropsychiatric diagnoses	
Specific Learning Disability	14 (12.8%)
Attention Deficit/Hyperactivity Disorder	9 (8.3%)
Speech or other Communication Disorder	4 (3.7%)
Intellectual Disability	3 (2.7%)
Tic or other Movement Disorder	2 (1.8%)
Autism Spectrum Disorder	1 (0.9%)
Mixed Specific Developmental Disorder	1 (0.9%)
Unspecified Psychiatric Disorder	1 (0.9%)

### Pandemic-related stressors

Table 2 shows the parent-reported problems related to the time of the pandemic.

**Table 2:** Pandemic-related stressors

Variables related to COVID-19 pandemic (parent-report)	N (%)
Infection with Sars-CoV-2 (affecting child and/or a family member)	1 (0.9%)
Quarantine (affecting child and/or a family member)	1 (0.9%)
Impact on parental job	
No access to previous occupation	24 (22%)
Layoff	23 (21.1%)
Dismissal	3 (2.7%)
None	60 (55%)
Impact on availability of basic supplies	
Lack of basic supplies	38 (34.9%)
More help received (e.g., food, vouchers)	52 (47.7%)
Impact on access to homeschooling	
Homeschooling from the beginning	74 (67.9%)
Homeschooling within one month	31 (28.4%)
Homeschooling beyond one month	4 (3.7%)
No homeschooling	7 (6.4%)
No homeschooling due to unavailability of technological devices	37 (33.9%)

Only one family reported Sars-CoV-2 infection and mandatory quarantine affecting a family member different from the minor interested in the survey. About half of the families underwent negative consequences on their jobs, mainly not managing to maintain previous occupation (22%) or being laid off (21.1%). 2.7% underwent dismissal. Many low-income families received some food or food vouchers (47.7%), but one-third of the sample experienced a lack of basic supplies (34.9%). Thirty-five people (32.1%) started homeschooling late, mainly within one month; seven children (6.4%) did not attend homeschooling at all. Thirty-seven families (33.9%) declared the impossibility of starting homeschooling promptly due to the unavailability of technological devices or a reliable internet connection.

### Major difficulties perceived by minors

Children and adolescents perceived their concern about health (either their own or that of their relatives and friends) as the major difficulty in 60.8% of the cases. More than half the sample declared that the impossibility to attend DSEC, due to its closure, was the greatest difficulty to sustain (55.7%). Other subjective difficulties were: school closure (37.1%), concern for the parental job (29.9%), impaired access to homeschooling (13.4%), living in a very small house (12.4%), lack of basic supplies (11.3%).

### Depressive symptoms and potential risk factors

At the CDI 2 PV, Emotional Problems subscale, 24/109 (22%) participants obtained scores above the cut-off, suggestive of typical depressive emotional symptoms (e.g., deflected mood, physical symptoms, negative self-esteem). Specifically, 6.4% showed above-average values (T-scores 60–64), 7.3% elevated values (T-scores 65–69), and 8.2% very elevated values (T-scores ≥ 70).

Those who answered the CDI 2 S-R SF were 100 minors, with 14% obtaining scores above the cut-off. Specifically, 3% showed above-average values (T-scores 60–64), 6% elevated values (T-scores 65–69), and 5% very elevated values (T-scores ≥ 70).

The correlations between potential risk factors for mental health and depressive symptoms were analyzed by logistic regression (Table 3). Because of insufficient data, we could not leverage all possible independent variables to compute the regression model (e.g., we had to exclude “Sars-CoV-2 infection” and “quarantine” as we reported only one positive case for both variables).

**Table 3:** Logistic regression analysis of factors influencing depressive symptoms assessed by CDI 2 Parent Version, Emotional Problems subscale

	Depressive Symptoms (Emotional Problems) (T ≥ 60) CDI 2 PV (*P*-value: 0.01239*)				
	Partial regression coefficient	Standard error	*P*	Odds Ratio (OR)	OR Confidence Interval 5%—95%
Pre-existing neuropsychiatric diagnosis	1.2225	0.524	0.020*	3.396	1.215—9.488
No access to previous occupation	-1.0051	0.738	0.173	0.366	0.086—1.555
Layoff	-0.4495	0.696	0.518	0.638	0.163—2.495
Lack of basic supplies	1.6177	0.686	0.018*	5.042	1.314—19.346
More help received (e.g., food, vouchers)	-1.2613	0.723	0.081	0.283	0.069—1.169
No homeschooling due to unavailability of technological devices	0.1381	0.585	0.813	1.148	0.365—3.610

*Note:* * indicates *p* < 0.05

Participants coming from families experiencing a lack of basic supplies were more expected to show depressive symptoms at CDI 2 PV, Emotional Problems subscale (OR 5.042; 95% CI 1.314—19.346; *p* < 0.05). Moreover, participants with a pre-existing neuropsychiatric diagnosis were more likely to exhibit depressive symptoms measured by CDI 2 PV, Emotional Problems subscale (OR 3.396; 95% CI 1.215—9.488; *p* < 0.05). The regression model considering the self-reported depressive symptoms as dependent variables remained above our a priori cut-off for statistical significance (*p* = 0.35).

## Discussion

Mental health is a critical concern in the pandemic scenario. It is particularly true when considering vulnerable populations exposed to greater environmental risk, like children and adolescents from socioeconomically disadvantaged households. We studied the psychopathological impact of the COVID-19 outbreak and pandemic-related stressors on a sample of Italian minors with socioeconomic disadvantage. In Italy, 1.3 million children and adolescents (13.5% of the whole minor population) suffered from poverty in 2020, and those living in the southern regions had the higher rates (14.5% versus 9.5% for the Center of Italy) [21]. In Southern Italy, the risk of food, school, and educational poverty is higher than in other Italian regions because: (1) there are the highest levels of failure to acquire minimum academic skills and school absenteeism; (2) the territorial supply of full-time school and school canteen is more scarce; (3) children and adolescents have the highest lack of sport, recreational, and cultural activities [22, 23]. All these factors affect children’s development and wellbeing from their earliest years and can have negative health and social consequences throughout their lives. We considered it important to focus our study on a population supported by DSECs because socioeconomically disadvantaged and so highly exposed to these environmental risk factors, hypothesizing that more acute disadvantages experienced during the pandemic would have had immediate consequences on the risk of developing depressive symptoms.

We measured depressive symptoms after the first wave of the COVID-19 pandemic, when mental health symptoms were expected to increase more than the previous acute phase, due to the emerging recession, according to the literature [2]. In our sample, 22% of subjects reported depressive symptoms measured by CDI 2 PV, Emotional Problems subscale; 14% of the minors fulfilling the CDI 2 S-R SF obtained elevated scores, suggesting depressive symptoms. None of them reported a pre-existing diagnosis of a depressive disorder: we can hypothesize that depressive symptoms could have recently emerged in all subjects. It is known that Major Depressive Disorder (MDD) can occur more frequently in individuals with a pre-existing genetic or environmental vulnerability, like the socioeconomic disadvantage [14]. The National Institute for Mental Health estimated the prevalence of MDD, in the year 2020, in 17% of American people aged 12–17 years old [24]. Depression is also one of the most frequent negative emotional responses during an epidemic outbreak [5, 25]. Its prevalence was expected to increase during the COVID-19 pandemic, in all populations and particularly in young people, due to the imposed restriction of social contacts [5]. Several large-scale studies from China provided reports of depressive symptoms in children and adolescents of the general population during the COVID-19 outbreak [25–29]. They reported a prevalence of depressive symptoms ranging from 11.78% [29] to 43.7% [25], based on single questionnaires. Our data are in line with this recent literature. Notably, for a subset of children and youth, the impact on the mental health of pandemic-related stressors will not be detected immediately but after some period of development [30]. Future epidemiological large-scale studies will reveal if the prevalence of MDD has actually increased among children and adolescents, as expected due to the growing environmental pandemic-related risk.

To date, to the best of our knowledge, no study had assessed the risk for mental health among socioeconomically disadvantaged children and adolescents during the COVID-19 outbreak. We found that almost half of the sample met conditions of more marked indigence during the pandemic. Nearly 50% of the households underwent worsening working conditions; 34.9% experienced a lack of essential supplies; 33.9% did not manage to access their children to homeschooling due to the absence of devices or Internet connection. Participants from families experiencing a lack of basic supplies were more expected to show depressive symptoms at CDI 2 PV. Poverty is one of the social determinants of general and mental health; additional factors introduced by the pandemic seemed to exacerbate the pre-existing vulnerability, making some poor even poorer [31]. It is known that socio-cultural and economic contexts significantly affect the prevalence of depression [32, 33]. We may consider the deficit of essential supplies as an extreme form of poverty, hitting low-income families who relied on educational institutions for providing healthy meals for their children. With the absence of this kind of help, several families were left alone in sustaining the nutrition needs of minors. Our data suggest that those who experienced a paucity of basic supplies had a higher risk to develop depressive symptoms at the end of the Italian first wave of the COVID-19 outbreak. It is possible to hypothesize that the increased environmental risk could trigger MDD in vulnerable individuals.

More than half the minors of the sample declared that the impossibility to attend DSEC, due to its closure, was the greatest difficulty to sustain. We can deduce that this kind of educational institution would be perceived as really supportive by most minors who benefit from adequate nutrition, educational support in learning activities, playgrounds for physical activities, and regular social contacts with peers and caring adults. It is clear how a perceived supportive environment could advantage the maintenance of the psychological balance in vulnerable people.

Another relevant issue is related to our findings: 26.6% of the sample declared a pre-existing neuropsychiatric diagnosis; all but one were neurodevelopmental disorders (NDDs), mainly represented by SLD and ADHD. This high prevalence is consistent with data from the 2016 National Survey of Children’s Health, where children living in lower-income households had a higher prevalence of a parent-reported diagnosis of mental, behavioral, or developmental disorder than children living in higher-income families (22.1% versus 13.9%) [34]. There is a lack of articles on children and adolescents with NDDs during the current pandemic, apart from Autism Spectrum Disorder (ASD) and ADHD [35]. In our sample, a pre-existing NDD correlated with the occurrence of depressive symptoms measured by the CDI 2 PV, acting as a further risk factor for depressive symptoms besides the socioeconomic disadvantage. As expected, children and adolescents with pre-existing neuropsychiatric problems were more susceptible to new mental health conditions in a time of crisis, like the present pandemic scenario [2, 36, 37]. That could be explained by several stressors, like the absence of school or other daily routines, that are important coping mechanisms for young people with NDDs like ADHD and ASD, or the inability to access regular care or mental health support [35, 37, 38]. SLD and ADHD, the most frequent neuropsychiatric diagnoses of our sample, can both hurt self-esteem, self-efficacy, and psychological well-being, favoring the development of internalizing symptomatology like anxiety and depression [38, 39]. We can suppose that children and adolescents with learning disorders (12.8% of our sample) suffered from the increased emotional distress of adapting themselves to online classes without adequate specific tools for addressing their learning needs [39, 40].

Following the literature, in our survey, the group of families who had children with pre-existing NDDs underwent a high burden of stress: they had to sustain the special educational needs of their children without any training and in the absence of external supporting networks [35, 37]. This kind of stress, combined with the higher economic pressure hitting low-income households, especially in times of crisis, raised the risk of increased domestic violence, which is considered one of the biggest threats to children’s mental health [41, 42].

The results of our study may be exemplary of a particular group of frail subjects represented by socioeconomically disadvantaged children and adolescents. They were more vulnerable to depressive symptoms during the COVID-19 outbreak, especially if they experienced a lack of essential supplies during the pandemic or had a pre-existing neurodevelopmental diagnosis.

The novelty of our study comes from focusing on a frail population in terms of socio-economic and healthy conditions, in a territory where the opportunity to heighten own social position is poor, with a consequent chronic high risk for health [43]. Socio-political interventions are needed on social inequalities which easily become health inequalities with an increasingly heavier economic burden.

Based on our findings, we can say that mental health promotion can benefit from fighting against food poverty, by broadening the opportunity of free healthy meals in schools and other educational institutions and giving vouchers or other forms of feeding sustain to needing people. Furthermore, access to care should be increasingly guaranteed for those with an NDD, thus reducing the risk of psychiatric comorbidities. Socio-political and health interventions are necessary to compensate for the detrimental effects of the pandemic in disadvantaged individuals. Socio-educational institutions and mental health services for children and adolescents must work synergically to detect and intervene promptly in case of need of psychological or psychiatric support [44]. In the hypothesis of another pandemic wave, we must be ready to adopt functional strategies to avoid the harmful effects of social isolation and economic recession in disadvantaged environments. Further studies are needed to detect effective preventive and therapeutic strategies for this purpose.

This research presents some limitations that should be highlighted. First, given the cross-sectional nature of data, it was not feasible to use the correlations for causal inferences. The second one was the small sample, which makes findings more difficult to generalize: although the required sample size was reached and overcome, it had been calculated with a relatively wide margin of error (10%). The low representativeness of the sample possibly derives from low compliance expectable from a population with a poor socio-cultural background. Future research could enhance the mediation of DESC’s staff in promoting participation in the survey because they are in direct touch with the families of the minors attending the service. Furthermore, it could be useful to widen the research on homogeneous samples recruited in other parts of Italy, to study the geographic distribution of socioeconomic risk factors for depression. Third, a selection bias was introduced because less than half of the invited DSECs participated in the survey, making the study sample not really representative of the population attending the DESCs of the territory. Furthermore, we could not describe which families participated in the study and which did not. Fourth, the association between depressive symptoms and risk factors resulted from the only parent-reported CDI 2 because the self-reported data were not statistically significant.

We think the main strength of our study was the assessment of the COVID-19 impact on mental health among a homogeneous vulnerable group represented by socioeconomically disadvantaged minors. The present research could have some beneficial long-term effects by identifying mechanisms (e.g., coping strategies, preventive interventions) to support vulnerable groups such as young people with low socioeconomic status. Future larger-scale studies among similar samples of vulnerable people could confirm our findings and provide new insights.

## Conclusions

The COVID-19 pandemic appears to have increased the already elevated risk for mental health affecting socioeconomically disadvantaged children and adolescents. The experience of a lack of essential supplies during the pandemic and pre-existing neurodevelopmental diagnoses were significantly associated with the occurrence of depressive symptoms in minors with a socioeconomic disadvantage at the end of the Italian first wave of COVID-19. International politicians should carefully consider vulnerable populations like children and adolescents from low-income households, who had to suffer the consequences of economic recession and the absence of support outside the family. The promotion of educational and child-care programs and activities could sustain the prevention of mental distress. There is no health without health promotion.

## Data Availability

The datasets used during the current study are available from the corresponding author on reasonable request.

## Abbreviations

COVID-19: Coronavirus Disease 2019
DSEC: Diurnal Socio-Educational Center
CDI 2 PV: Children’s Depression Inventory 2 Parent Version
CDI 2 S-R SF: Children’s Depression Inventory 2 Self-Report Short Form
SLD: Specific Learning Disability
ADHD: Attention-Deficit/Hyperactivity Disorder
MDD: Major Depressive Disorder
NDD: Neurodevelopmental Disorder
ASD: Autism Spectrum Disorder
